# The Impact of COVID-19 Pandemic on Seafood Safety and Human Health

**DOI:** 10.3389/fmicb.2022.875164

**Published:** 2022-06-22

**Authors:** Nikheel Bhojraj Rathod, Nariman Elabed, Fatih Özogul, Joe M. Regenstein, Charis M. Galanakis, Sulaiman Omar Aljaloud, Salam A. Ibrahim

**Affiliations:** ^1^Department of Post Harvest Management of Meat, Poultry and Fish, Post-graduate Institute of Post-harvest Management (Dr. Balasaheb Sawant Konkan Krishi Vidyapeeth), Raigad, India; ^2^Laboratory of Protein Engineering and Bioactive Molecules (LIP-MB), National Institute of Applied Sciences and Technology (INSAT), University of Carthage, Carthage, Tunisia; ^3^Department of Seafood Processing Technology, Faculty of Fisheries, Cukurova University, Adana, Turkey; ^4^Department of Food Science, Cornell University, Ithaca, NY, United States; ^5^Research and Innovation Department, Galanakis Laboratories, Chania, Greece; ^6^Food Waste Recovery Group, ISEKI Food Association, Vienna, Austria; ^7^College of Sports Science and Physical Activity, King Saud University, Riyadh, Saudi Arabia; ^8^Food Microbiology and Biotechnology Laboratory, 171 Carver Hall, College of Agriculture and Environmental Sciences, North Carolina A & T State University, Greensboro, NC, United States

**Keywords:** COVID-19, SARS-CoV-2, seafood safety, natural antimicrobials, functional foods, seafood processing

## Abstract

The coronavirus disease (COVID-19) pandemic caused several negative impacts on global human health and the world’s economy. Food and seafood safety and security were among the principal challenges and causes of concern for the food industry and consumers during the spread of this global pandemic. This article focused on the effects of COVID-19 pandemic on potential safety issues with seafood products and their processing methods. Moreover, the potential impacts of coronavirus transmission through seafood on human health were evaluated. The role of authenticity, traceability, and antimicrobials from natural sources to preserve seafood and the possible interaction of functional foods on the human immune system are also discussed. Although seafood is not considered a principal vector of SARS-CoV-2 transmission, the possible infections through contaminated surfaces of such food products cannot be neglected. The positive effects of seafood consumption on possible immunity built up, and COVID-19 are also summarized.

## Introduction

A zoonotic virus causes coronavirus disease (COVID-19) (novel coronavirus) usually originating from bats that cause severe acute respiratory syndrome-coronavirus 2 (SARS-CoV-2). It has rapidly spread worldwide from the first report of pneumonia cases in December 2019 and was declared a pandemic in March 2020 by the WHO (Tian et al., 2020). Negligible risk of contamination in foods due to contact with infected persons through hands, sneezing and coughing, or contaminated raw material and risk associated with the supply chain are considerable (Hakovirta and Hakovirta, 2020; Rizou et al., 2020). Such scenarios highlight food safety issues due to handling infected material and its unintentional person-to-person spread by food handlers in all food sectors (Shahbaz et al., 2020).

Furthermore, coronavirus 2 is proven to undergo mutation (Delta and Delta plus) on the spike protein (D614G), providing them protection against the vaccine, antibody therapeutics, and increasing pathogenicity in some cases, SARS-CoV-2 is no different (Korber et al., 2020; Kannan et al., 2021). The newest COVID-19 strain was reported to spread much rapidly, spreading across 210 countries in about a month (Harvey et al., 2021; Zeyaullah et al., 2021), enhanced replication in human lungs (Plante et al., 2021), further increasing the transmission. The mutation is reported to change amino acid positions, improving the binding affinity of the spike protein (Abdool Karim and de Oliveira, 2021). Therefore, the possibility of reduced vaccine-induced immunity, while natural immunity proved more stable to infection with replicated variants, is possible (Abdool Karim and de Oliveira, 2021; Harvey et al., 2021).

The COVID-19 pandemic has caused many countries’ total or partial closure due to the rapid spread of the virus and higher mortality rate, hampering businesses with an increase in work from home (Hamadani et al., 2020). However, many food-related activities are impossible to do from home, directly exposing the food sector to COVID-19. In addition, the pandemic has emphasized the need for optimum nutrient uptake with a role in building immunity (de Faria Coelho-Ravagnani et al., 2021). Nevertheless, severe food insecurity and lowered consumption of nutritionally rich foods were reported during the pandemic in African countries (Kansiime et al., 2021). Considering the association of COVID-19 from the seafood market, the consumption and trade related to seafood were feared in the early days of the pandemic (Eftimov et al., 2020; Meharoof et al., 2020; White E. et al., 2021; Zhang et al., 2021). However, SARS-CoV-2 was not considered to be foodborne. However, the impact of the pandemic on the food sector was dramatic and highlighted the fact that our food systems are fragile and need a transformation led by disruptive technologies and reconsideration of our consumption norms (Galanakis, 2020; Galanakis et al., 2021). Raw or undercooked foods of animal origin are not recommended as a precautionary measure. Fish and meat are rich sources of glucosaminoglycans which could anchor and transmit COVID-19 (Pressman et al., 2020). However, the high nutritional content of fish (fish oil, vitamins, mineral) are helpful as potential inhibitors and adjuvants as part of COVID-19 therapy (Ahern et al., 2021; Torrinhas et al., 2021) making the inclusion of fish potentially beneficial for COVID-19 quarantine (Abdool Karim and de Oliveira, 2021) and quality proteins from fish are known for their ability to strengthen immunity. With novel technologies being under evaluation against COVID-19 in seafood has been recently reviewed by Kulawik et al. (2022). It also covers the effects of processing and preservation methods using natural antimicrobials on COVID-19. Finally, the potential role of seafood and natural ingredients on human health, improving immunity, and reducing the risk of COVID-19 are presented.

## Seafood Authenticity and Traceability Concerns During COVID-19

Due to the globalization of the seafood market and modernization of the fishing industry, adulteration or substitution of cheaper and lower grade fish is a growing problem. Items may belong to entirely different species and have another place of origin, suggesting the importance of greater authenticity and traceability in the seafood industry. Post-processing, it is challenging to identify a skinless portion of fish flesh based on its morphology (Freitas et al., 2020). As seafood consumption has been associated with several food poisoning and seafood-borne illness cases in the past, authenticity and traceability are needed to manage food safety risks (Freitas et al., 2020).

Authenticity in seafood means providing detailed information on the species, origin/capture, wild or cultivated, and about any genetic modifications, if any. These must be detailed when exporting to the European Union countries, with one of the most comprehensive standards for seafood (Hofherr et al., 2016).

Methods to determine fraud include changes in fatty acids, proteomic composition, and elemental profiles (Lavilla et al., 2013). Other research studies have also provided different advanced methods. There is also a need to validate this information (Freitas et al., 2020).

Traceability can identify the exact path through the harvest area, production site, the fish’s identity and any catch certificate, the handling, food safety processing operations and standards, supply chain issues, and different regulatory protocols (Freitas et al., 2020). In addition, consumers are at risk as the substitution of fish has been related to the transmission of several zoonotic parasites, which may have several health risks (Attwood and Hajat, 2020).

## Seafood Security and Safety Concerns With COVID-19

Due to lockdowns enforced by some governments, entire food supply chains were forced to shut down. Production, harvesting, processing, and marketing were severely hampered; fish/seafood was no different. As the COVID-19 originated from a seafood market, consuming all forms of meat declined as a precautionary measure, especially undercooked products (Ahmadiara, 2020; Bassett et al., 2021; White K. et al., 2021). The COVID-19 pandemic was also related to the closure of some processing plants in the United States dealing with meat and seafood (Centers for Disease Control and Prevention, 2021). Later research confirmed that infections were only found in mammals (Lam et al., 2020). However, seafood trade-related activities were reduced during the pandemic (Stoll et al., 2021). Several earlier reports on coronavirus have been reported to be stable at ambient and frozen temperature, increasing the associated risks (Tokur and Korkmaz, 2021). Also, the high stability of SARS-CoV-2 for extended periods (2 weeks) at chilled and frozen temperature conditions have been confirmed (World Health Organization, 2020).

Survival of SARS-CoV-2 at 4°C attached to salmon culture media, exploring the possibilities of transmission (end point titration assay on Vero E6 cells) in seafood markets was evaluated by Dai et al. (2020). Results proved the viability of the virus to remain active for 8 days at 4°C. The study highlights the requirement of strict regulations in the seafood trade due to the provision of lower temperatures in the seafood supply chain.

Food security and safety are always considered in any food supply channel, especially during a pandemic (Ma et al., 2021). Several modern and traditional methods for virus inactivation have been discussed by Ahmadiara (2020), such as high-temperature heating, irradiation, ultraviolet light, and high-pressure processing and using antiviral agents for disinfecting. Apart from the destruction of microorganisms, several issues focusing on food safety include the health status (e.g., sick) of those handling the product, social distance, personal hygiene (e.g., washing hands), using latex gloves, and avoiding cross-contamination from utensils and equipment using proper disinfection (Rizou et al., 2020) to prevent spreading the pandemic (Rizou et al., 2020). The intervention of many workers’ hands in fish harvesting and processing can cause a high spread of COVID-19 (Havice et al., 2020).

**Figure 1:**
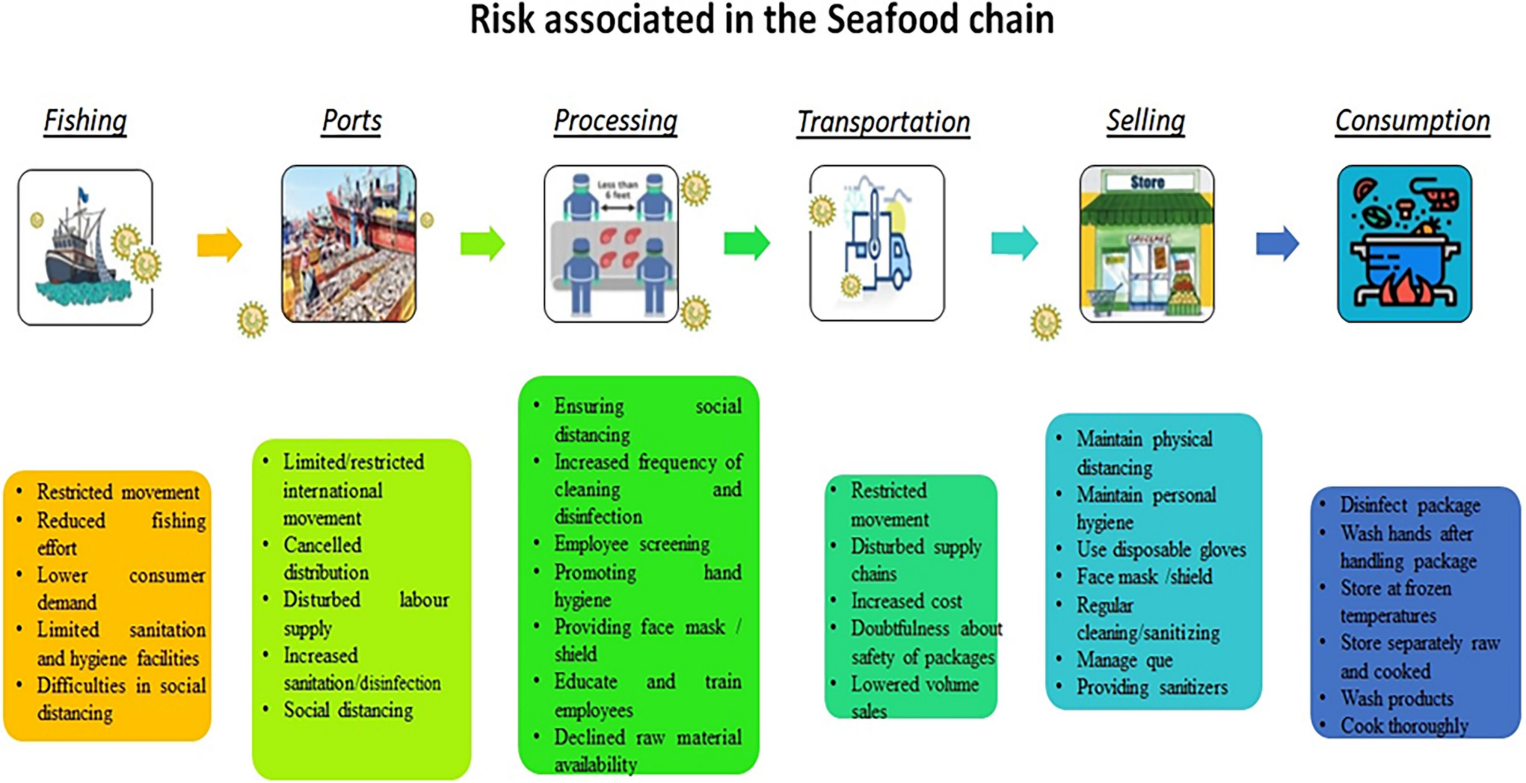
Risks associated in the seafood chain.

Transmission of coronavirus through fresh or processed seafood has not been reported. However, the possible transmission could be through cross-contamination from infected persons over the surfaces or packaging materials, further spreading by consuming contaminated food or by contact with the contaminated package (Rizou et al., 2020). Several risks associated with the transmission of SARS-CoV-2 through seafood chains are shown in Figure 1.

## The Effect of COVID-19 on Processing Methods for Seafood

Seafood and its products are essential because of the richness of their essential micronutrients, including several vitamins, dietary proteins as well as omega-3 fatty acids, principally long-chain ω-3 polyunsaturated fatty acids (LC n-3 PUFA), such as docosahexaenoic acid (DHA) and eicosapentaenoic acid (EPA), which are beneficial for humans (Inanli et al., 2020). In addition, antiviral compounds from marine resources have been evaluated and found helpful in COVID-19 treatment (Zahran et al., 2020; Geahchan et al., 2021). However, seafood quality can be decreased due to enzymatic, chemical, and microbiological deterioration, which can cause several safety issues (Inanli et al., 2020). Thus, these products should be preserved using different preliminary processing methods (e.g., washing and filleting) to assure their eventual quality and safety after additional processing. In addition, some recent studies have highlighted the potential of transmission of SARS-CoV-2 through the animal food product supply chain (Fu et al., 2021; Hu et al., 2021).

Many processing methods are used with seafood to inhibit or delay the growth of spoilage and pathogenic microorganisms and inactivate the alterations due to the enzymatic reactions, including freezing, chilling, packaging using a modified atmosphere (MAP), vacuum packaging, irradiation, heating, salting, smoking, and drying (Boziaris, 2014) to assure the safety and stability of seafood as well as to enhance its shelf life. Hopefully, with minimal unfavorable effects on their organoleptic attributes and the number of essential nutrients. Seafood processing methods using lower temperatures, such as freezing and chilling, are the most applied treatments to conserve seafood and its by-products. Moreover, the application of packaging methods (e.g., MAP) can extend the seafood shelf life by delaying microbial growth and decreasing oxidative reactions (Boziaris, 2014). Heating and irradiation methods are also used to inhibit the growth of spoilage and pathogenic microorganisms, reduce chemical oxidative stress, and prevent enzymatic browning in seafood (Boziaris, 2014).

However, the widespread outbreaks involving several new microorganisms, including COVID-19, impose a new challenge to global nutrition and food safety (Love et al., 2021; Nakat and Bou-Mitri, 2021). Therefore, the food and seafood sectors have required an increased focus on preventive and precautionary measures. In addition, several contradictory reports about the direct transmission of SARS-CoV-2 to humans through foods have been reported. The Centers for Disease Control and Prevention (CDC) in the United States and the WHO have rejected food transmission, while a recent study by Hu et al. (2021) suggested the possibility of increasing the infection with this virus by the consumption of contaminated food.

SARS-CoV-2 viral RNA can survive on seafood and other food products at 4°C for more extended periods than MERS-CoV RNA (Godoy et al., 2021), and SARS-CoV-2 has been found on seafood (White K. et al., 2021). As a result, SARS-CoV-2 has had huge impacts on the seafood sector, such as processor closings, shortening of fishing seasons, and disruptions in production and distribution of seafood (White E. et al., 2021) with less impact on frozen products (White K. et al., 2021).

**Figure 2:**
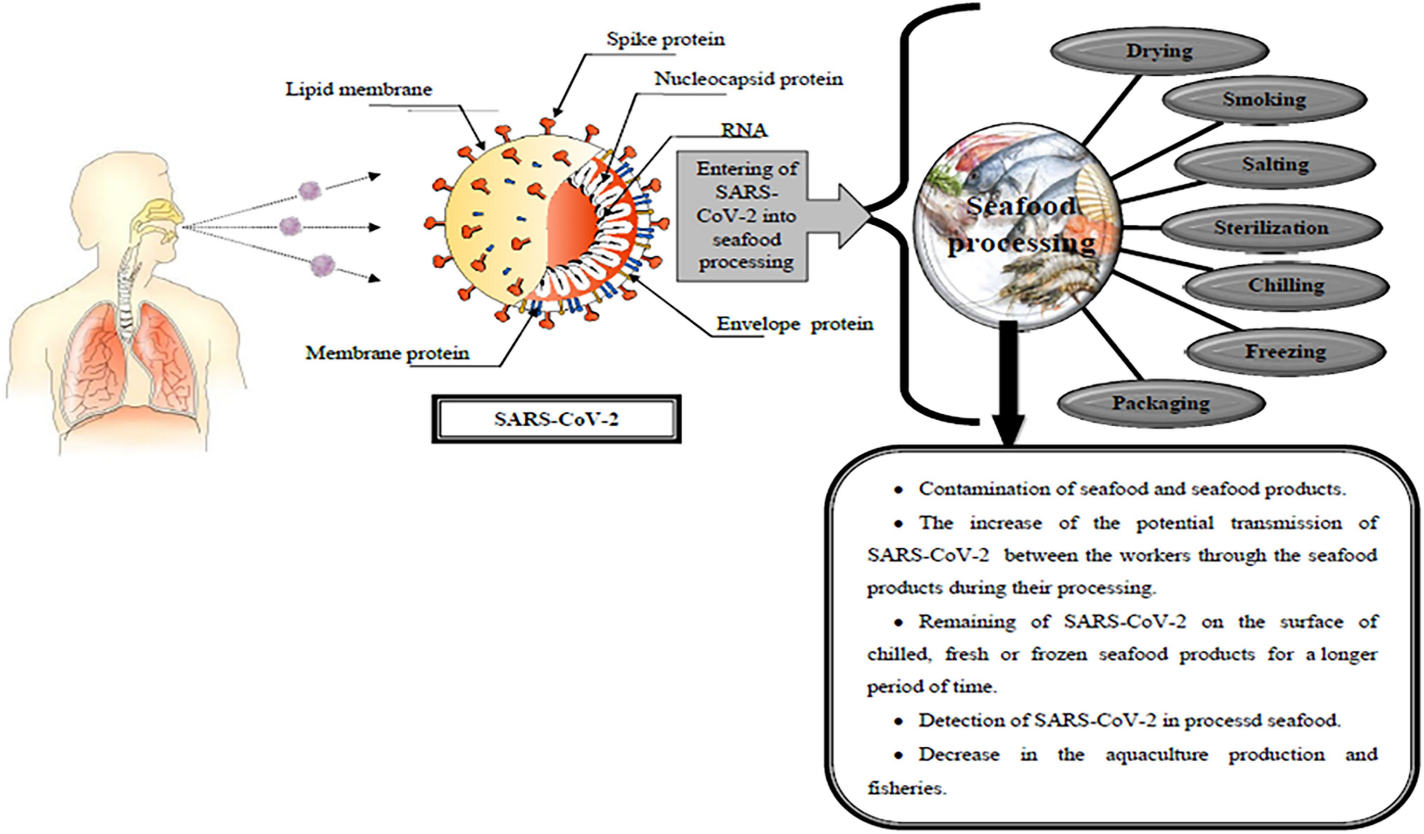
The impacts of SARS-CoV-2 on seafood processing methods.

During the COVID-19 pandemic, food products were contaminated with SARS-CoV-2, particularly meat and seafood products, which can transmit this virus during seafood processing (Chitrakar et al., 2021). In addition, seafood workers can be contaminated with COVID-19 due to the virus spreading during extended periods of confined work (White E. et al., 2021). Fresh seafood can also be contaminated by infected persons who prepare or serve seafood at home or in restaurants (Duda-Chodak et al., 2020). On the other hand, SARS-CoV-2 can be transmitted through contact with fish products contaminated and stored at different temperatures: 4°C, −20°C, and −80°C due to the ability of the virus to survive on the surface for 3 weeks in all of these samples (Ceniti et al., 2021). The impact of SARS-CoV2 on seafood processing methods is shown in Figure 2.

### Fresh Seafood

Fresh seafood is the most preferred form (Nguyen et al., 2015). Fresh seafood can be sold in different forms depending on the species (e.g., whole, gutted, headed and gutted, steak and fillet with skin on or off; Nguyen et al., 2015). Unfortunately, the pandemic has led to a decrease in fresh sales in many countries and a 30%–40% decrease in exports (White E. et al., 2021). Thus, the pandemic has strongly negatively impacted communities dependent on seafood products.

The virus can remain on the surface of chilled fresh seafood, such as salmon, for more than a week, increasing the international transmission of this virus. Thus, the experimental studies have confirmed that the SARS-CoV-2 can survive on seafood products for 8 days at 4°C (Ceniti et al., 2021). Another study showed that SARS-CoV-2 could survive on fish for more than a week in refrigerated rooms at 4°C (Godoy et al., 2021). The SAS-CoV-2 can also remain viable on salmon for 8 days at 4°C and 2 days at 25°C (Han S. et al., 2021). Thus, contaminated seafood products in the cold chain with coronavirus can be considered the amplifying source of the virus through transport over long distances (Han J. et al., 2021), although most public health agencies have confirmed that food is not a vector for the transmission of the virus (World Health Organization, 2019).

### Frozen Seafood

Freezing has a vital role in inhibiting the growth of diverse microbial species and generally reduces the chemical reactions in the food matrix. Hence, freezing can extend the shelf life of food products.

However, SARS-CoV-2 has been found in frozen seafood during processing. For example, this virus has been detected on the packaging materials of frozen shrimp (Chitrakar et al., 2021) and survives for a long time when frozen, as described above. Another study showed that both frozen (−20°C) and refrigerated (4°C) salmon samples after 3 weeks retained the virus. In Beijing’s Xinfadi market, SARS-CoV-2 was found on salmon surfaces (Liu et al., 2020). This led to China examining all imported seafood for SARS-CoV-2 RNA (Godoy et al., 2021).

### Other Processing Methods

Although both thermal processing (such as pasteurization, sterilization, and high-temperature drying) or non-thermal processing methods (such as ionizing radiation, ultraviolet light, high-intensity ultrasound, and high hydrostatic pressure) are used to extend shelf life and preserve seafood, the quality and safety of these products concerning COVID-19 needs to be considered. The transmission of COVID-19 between seafood workers raised concerns about the safety of aquatic food products. Thus, the seafood sector has suffered recently from a systemic problem due to the COVID-19 pandemic. Besides finding the virus on the product, at one seafood market, the virus was found on the cutting boards used to process the imported seafood, e.g., salmon (Olatunde et al., 2021). Contamination can be through respiratory droplets from infected humans or the contact between their hands and foods, leading to problems for the industry (Ayseli et al., 2020).

China has suspended shrimp import from three Ecuador producers because of the discovery of traces of SARS-CoV-2 on the external packaging of six samples (Godoy et al., 2021). In addition, the epidemiological studies suggested that COVID-19 spread in Qingdao was apparently due to the contamination of cod with SARS-CoV-2 at some point (Liu et al., 2020).

## The Effects of COVID-19 on Human Health Concerning the Consumption of Seafood

COVID-19 is known to induce high-grade fever, tiredness, and increased coughing 1 to 14 days after someone becomes infected (Singh et al., 2020). The lockdowns to limit the spread of COVID-19 has hampered the capture and movement of seafood (Ma et al., 2021). Love et al. (2021) have discussed the issues hampering the entire seafood supply chain during this pandemic. Several studies have questioned the spread of COVID-19 through food, especially muscle foods (meat and fish), due to their lowered temperature of processing and storage conditions (Han S. et al., 2021). Han J. et al. (2021) reported the stability of COVID-19 on the skin of muscle foods for 14–21 days, which is a matter of concern. Alternatively, Bondad-Reantaso et al. (2020) ultimately rejected the transmittance of COVID-19 through seafood. Immunity factors have become important by imparting resistance against viral infections. However, dietary supplements and nutraceuticals from aquatic sources have been evaluated for preventing and treating COVID-19 (Das, 2021; Yu et al., 2021).

Furthermore, consuming 26 pounds of seafood per person *per annum* is recommended to derive the benefits (U.S. Department of Agriculture and U.S. Department of Health and Human Services, n.d.). Seafood is rich in polyunsaturated fatty acids, proteins, bioactive compounds, anti-inflammatory, vitamin-D, B6, and micronutrients. Further, the virus exhibited several variations, imparting them enhanced transmissibility, causing more severe illness, and reduced neutralization of viruses by vaccines impacting treatments (Harvey et al., 2021; Zeyaullah et al., 2021). The negative impact of diet, depression, and obesity, along with the protective effect of seafood consumption on the fatality of COVID-19 patients, was reported (Rajkumar, 2021) based on bivariate analysis of COVID-19 mortality data from 156 countries. Therefore, they are recommending an increase in seafood consumption in COVID-19 patients.

The role of adequate fatty acids and their metabolites on the inactivation of SARS-CoV has been described by Das (2021). Seafood is regarded as one of the most promising sectors with major international trade. Belluzzi et al. (2020) highlighted the immunity improving ability of EPA, which overcomes the immune-suppressing effects of medication. Yu et al. (2021) evaluated the bioactive components derived from tuna proteins degraded to form peptides and showed their antiviral effects when used as a nutritional supplement. It was a strong SARS-CoV-2 inhibitor. These peptides were safe with good solubility. However, only one peptide showed docking with the virus-host with 144 Kcal/mol energy value.

Selenium, an essential micronutrient found in seafood, has been reported to increase immunity, increase vaccine response, and reduce inflammation (Avery and Hoffmann, 2018). All of these factors are associated with the current COVID-19 pandemic. Das (2021) suggested the correct proportion of arachidonic acid, EPA, and docosahexaenoic acid could benefit COVID-19 infections. In addition, zinc is associated with an improved immune system and the inactivation of viral infections (Jayawardena et al., 2020).

The marine secondary metabolite, polyphosphate (polyP 10 μg/ml) from marine bacteria, exhibited a strong antiviral impact on SARS-CoV-2 reduced binding by 42% with cellular receptors by inducing innate immunity and improving mucin barrier. Furthermore, the effect was found to increase with the concentration of polyphosphate (Müller et al., 2020). Inorganic polyphosphate in the form of nanoparticles (magnesium salt of inorganic polyphosphate) with plant metabolite (quercetin) against COVID-19 was evaluated (Neufurth et al., 2021). The combination was said to act as an antioxidant and increased the *MUC5AC* gene responsible for clearing the lung. Further, polyP could inhibit spike protein by acting on the receptor-binding domain (Geahchan et al., 2021).

Vigorous antimicrobial activity of *Abalone viscera* against SARS-CoV-2 pseudovirus was exhibited IC50 33 μg/ml (Yim et al., 2021). Gentile et al. (2020) screened (14,064 compounds) and reported 17 potent protease inhibitors against SARS-CoV from marine origin. The selection was confirmed based on structure and ligand-based drug design approach. Cobre et al. (2021) proved the protective (GLM based) effects of seafood, their proteins, and lipids in recovery against COVID. Finally, Sumon et al. (2021) reviewed peptides originating from aquatic organisms for their antiviral potential for application as therapeutics. The review discussed different aquatic antiviral peptides and their ability to induce immune pathways and highlighted their ability to inhibit SARS-CoV-2 through blocking their entry, replication, and release mechanism.

Aatif et al. (2021) reported ACE2 blocking by receptor binding domain using marine seaweed. Dieckol (found in brown alga *Ecklonia cava*) derivative (DK07) exhibited higher receptor binding ability. The derivative (DK07) showed interaction with crucial amino acids against the mutated United Kingdom variant (VUI-202012/01) of SARS-CoV-2. Yim et al. (2021) reported the inhibition of SARS-CoV-2 by sulfated fucoidan and crude polysaccharides obtained from seaweed species (*Undaria pinnatifida* sporophyll, *Laminaria japonica*, *Hizikia fusiforme*, *Sargassum horneri*, *Codium fragile*, *Porphyra tenera*). Seaweed polysaccharides exhibited high molecular weight (>800 kDa), carbohydrate (62.7%–99.1%), fucose (37.3%–66.2%) content, and antiviral activities against SARS-CoV-2 pseudovirus, except *P. tenera*. The high antiviral ability was the synergistic effect of bioactive compounds and high molecular weight polysaccharides. The recent review by Fitton et al. (2021) reviewed the role of fucoidan, found in seaweeds against respiratory viral infection (SARS-CoV-2). It clarified the ability of fucoidan to inhibit COVID infection, increase immunity, and enhance inflammatory response. Antiviral activities of purified sulfated polysaccharide and lambda-carrageenan from marine red algae against coronavirus two were reported (Jang et al., 2021). The EC_50_ value of 0.9 ± 1.1 μg/ml and no toxic effects were observed up to a 300 μg/ml concentration. Further, reduced expression of virus proteins and progeny production were observed in a dose-dependent manner.

Plitidepsin from tunicate (*Aplidium albicans*) was reported to inhibit SARS-CoV-2 (IC_90_—1.76 nM), inhibition in human cell line was much effective IC_90_—0.88 nM, which was over commercially used remdesivir (White K. et al., 2021). Additionally, inhibition of pneumocyte-like cells was also observed IC_90_—3.14 nM, and replication was also inhibited (White K. et al., 2021).

Aquatic organisms and processed seafood are regarded as potential routes for the transmission of pathogenic microorganisms (Fu et al., 2021). Seafood processing requires special processing facilities and maintenance of the cold chain. However, basic hygienic practices are mostly inadequate in many seafood processing facilities (Cook et al., 2017), although no COVID-19 transmittance has been traced to a seafood purchase (Poelman et al., 2021).

## The Effects of Natural Antimicrobials and Functional Foods on the Immune System of Humans With COVID-19

The immune system has a significant role in defending against various infections (Hollman, 2001), including COVID-19 (Benarba and Pandiella, 2020), which became a powerful tool for resisting the COVID-19 disease (Ayivi et al., 2021). Natural antimicrobials from plants and microorganisms (fermented foods, prebiotics, probiotics, and synbiotics) enhance immunity by modulating gut and respiratory systems. They were discussed by Sultan et al. (2014) and Antunes et al. (2020) for their effectiveness against COVID-19. Plant source compounds consisted of immunomodulatory bioactive compounds, while those from microorganisms could be included in therapeutic strategies against COVID-19. Functional foods may improve health and support resistance to several viral infections and nutritionally related diseases (Rastmanesh et al., 2020). Functional foods are foods with one or more specialized functions in the body, usually obtained from naturally occurring components, including improvements in immunity (Han and Hoang, 2020). Galland (2013) suggested that bioactive peptides, fish oils, and fish protein hydrolysates could be functional foods derived from seafood. Mu et al. (2021) have reported that garlic and *Rhizome polygonati* are available against the SARS-CoV-2 virus. The increased immunity and antimicrobial activity of garlic could be used to prevent COVID-19 infection. Caffeic acid derivative-based functional foods could be used to develop anti-COVID-19 therapies (Adem et al., 2021). Selective and proper functional/bioactive material should be supplemented to enhance immunity (Ayivi et al., 2021).

Han and Hoang (2020) have discussed the role of different functional components/foods that could boost immunity and be used as probable components with the current pandemic. For example, vitamins A, B6, B9, B12, C, D, E, and zinc and iodine help regulate inflammation and protect against viral infections (Calder and Grimble, 2002). Conversely, the deficiency of any of the above nutrients can impair the maintenance of homeostasis (Ayivi et al., 2021).

**Figure 3:**
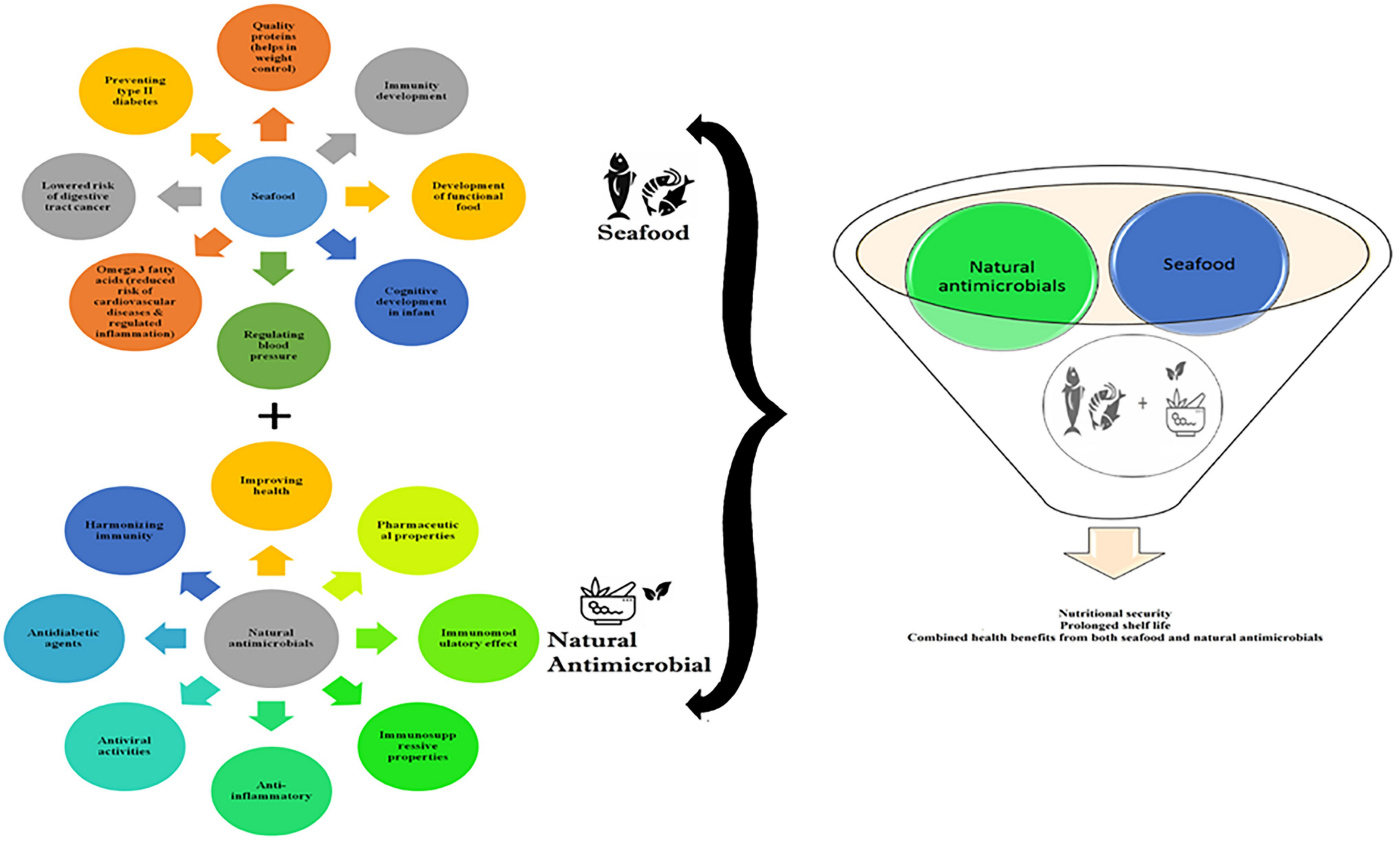
Health promoting benefits of seafood and natural antimicrobial.

Sultan et al. (2014) have discussed the bioactive components present in garlic, ginger, purple coneflower, black cumin, and liquorice to maintain homeostasis and boost immunity. Phenols, phenolic acids, carotenoids, flavonoids, and other bioactive phytoconstituents in natural plant compounds impart bio-activity and enhance immunity (Chojnacka et al., 2020; Surendran et al., 2020) and have antiviral activity against coronavirus (Nebigil et al., 2020; Rameshkumar et al., 2021). Al Jitan et al. (2018) outlined the different phenolic acids present in plants and their importance in the human diet. Phenolic acids suppress oxidation due to their antioxidant capacity, which is related to increased immunity. Additionally, phenolic content has been shown to suppress inflammation and regulate blood sugar levels (Li et al., 2014). Ding et al. (2018) studied the ability of polyphenols to bind receptors on immune cells and regulate the immune system. They reported improved intestinal immune responses and regulation of allergic diseases. Recently, Surendran et al. (2020) highlighted the plant metabolites that inhibit ACE activity relevant to the current pandemic situation while also lowering the risk of cardiovascular diseases along with some antiviral activity. The health benefits of seafood working with natural antimicrobials are shown in Figure 3. This could provide the effects of seafood discussed in “The Effects of Natural Antimicrobials and Functional Foods on the Immune System of Humans With COVID-19” section and natural compounds discussed in this section. The role of aquatic-based functional foods directly or indirectly associated with boosting immunity, providing resistance against COVID-19, is shown in Table 1.

**Table 1:** Impact of aquatic-based functional foods on immune system/COVID-19.

Functional component	Action	References
Protein hydrolysate	Immunomodulatory, antioxidant, and anti-hyperglycemic	Roslan et al., 2014; Nasri, 2017; Chakrabarti et al., 2018; Salem et al., 2018; Zamora-Sillero et al., 2018
Peptide	Antihypertensive: prevents activity of angiotensin-I-converting enzyme (ACE), antioxidant, and anti-inflammatory	Roslan et al., 2014; Adnan et al., 2017; Nasri, 2017; Balami et al., 2019; Cong et al., 2020; Durand et al., 2020; Narayanasamy et al., 2020
Lipids	Regulating oxidative stress and anti-inflammatory properties	Suleria et al., 2015; Kundam et al., 2018; Calder et al., 2020
Polysaccharides	Immunomodulatory, anti-inflammatory, antioxidant, and antidiabetic	Zhao et al., 2018; Arasukumar et al., 2019; Lakshmana et al., 2019; Sim et al., 2019

## Impacts of Natural Antimicrobials on Risk Associated With COVID-19

The emergence and rapid propagation of COVID-19 in such a short time have caused a high number of fatalities. As a result, some efforts have focused on the development of effective antivirals to combat COVID-19. Additionally, the risk of excessive use chemical and synthetic antibiotics may pose a future health threat by increasing the chances of developing antibiotic resistance (Usman et al., 2020). Natural antimicrobials (Plant origin turmeric rhizome, bay leaf, black pepper, oregano, Indian surge tree, red spider lilly, tonka bean, *Cinnamomum aromaticum* Nees, *Vitis vinifera*, Japanese apricot, honey, astaxanthin) are rich source of metabolites (essential oils, polyphenolic compounds, triterpenoids, alkaloids, saponins) used to treat several potentially fatal bacterial (*Staphylococcus* spp., *Streptococcus* spp., *Listeria monocytogenes*, *Enterococcus*) and viral infections (dengue virus, herpes simple virus, human coronaviruses, SARS-CoV, SARS-CoV-2). Additionally, they improve the action of antibiotics by acting as coadjuvant used to treat several potentially fatal viral infections (da Silva Antonio et al., 2020; López-Malo et al., 2020) and are being used against COVID-19 (Li et al., 2005; Chang et al., 2012; Nishide et al., 2019; Rosmalena et al., 2019; da Silva Antonio et al., 2020; Talukdar et al., 2020; Wang et al., 2020; Ali et al., 2021; Rathod et al., 2021a,b; Stan et al., 2021; Zannella et al., 2021). The presence of peptides, polyphenols, hydroxybenzoic acid, hydroxycinnamic acid, lignans, fatty acids, tannins, and alkaloids, have been tested due to their high bioavailability, low toxicity with antiviral abilities without development of resistance (da Silva Antonio et al., 2020; López-Malo et al., 2020; Nebigil et al., 2020). Several studies have focused on screening and application of naturally occurring antiviral compounds exhibiting diverse antiviral activity by restricting entry and replication of virus (Benarba and Pandiella, 2020; da Silva Antonio et al., 2020; Li et al., 2020; Wang et al., 2020).

The inhibitory activity is attributed to phenolics and their antioxidant potential (Sharifi et al., 2013). Natural antimicrobials, including peptides, polyphenols, hydroxybenzoic acid, hydroxycinnamic acid, lignans, fatty acids, tannins, and alkaloids, have been tested due to their high bioavailability and low toxicity (da Silva Antonio et al., 2020; López-Malo et al., 2020). These benefits of antiviral activity without leading to the development of resistance by various microbes in the gut microbiome (Nebigil et al., 2020). In addition, several papers have described the successful screening and applications of naturally occurring antiviral compounds that hamper the entry, attachment, and replication of the virus (Benarba and Pandiella, 2020; da Silva Antonio et al., 2020; Li et al., 2020; Wang et al., 2020).

### The Effects of Antimicrobials on COVID-19

Antimicrobials are substances capable of inhibiting/retarding, and restricting the growth of microorganisms. However, COVID-19 is an enveloped, symmetrical and single-stranded RNA virus (Khan et al., 2020). The entry of SARS-CoV requires an angiotensin-converting enzyme (ACE) 2, followed by its cleavage by the transmembrane serine protease (Hoffmann et al., 2020) as a receptor and papain-like proteinase (Jiang et al., 2020) for replication of polyproteins. To date, chemical pharmaceuticals (host protein targeted, virus targeted, and nucleoside analogs) and traditional Chinese medicines have been administered to protect against COVID-19 by disturbing human host interaction (Li et al., 2020). Therefore, the primary active sites should be the structural and non-structural proteins.

**Figure 4:**
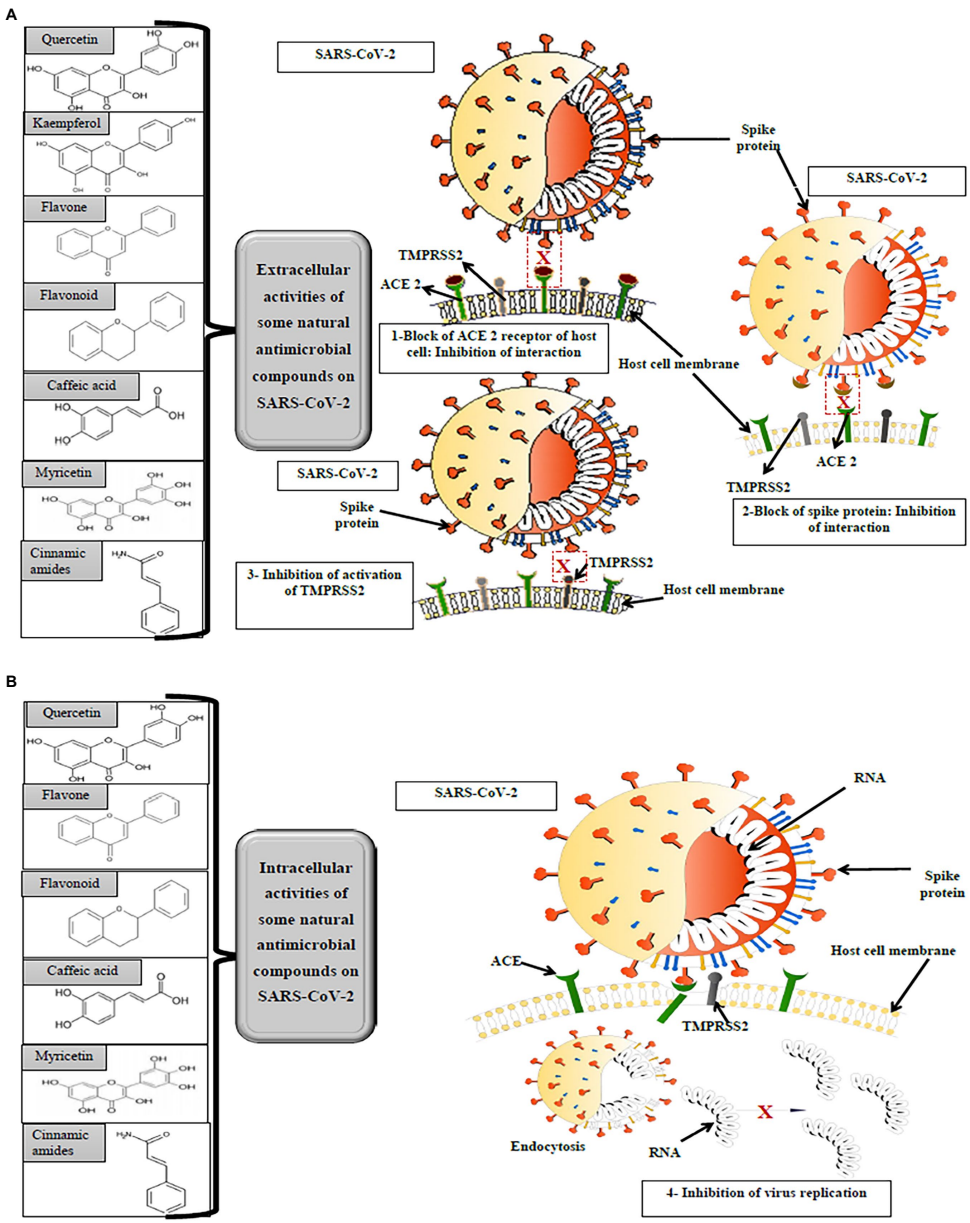
The different antimicrobial activities of some natural compounds against SARS-CoV-2.

A proposed action of antimicrobials is to block the ACE 2, transmembrane serine protease 2 (TMPRSS2), and target papain-like proteinase (PLPRO). Benarba and Pandiella (2020) discussed and justified the ability of natural products to block ACE 2, thus avoiding the spread and expression of COVID-19. The anti-transmembrane serine protease activity of natural kaempferol (Da et al., 2019) and flavonoids (Rameshkumar et al., 2021) have been reported. In addition, several natural compounds such as cinnamic amides and flavonoids have been shown to inhibit papain and chymotrypsin-like proteinases (Benarba and Pandiella, 2020). Figure 4 shows the action of some natural compounds against SARS CoV2.

### Natural Antimicrobials (Plant Source) Against COVID-19

Plants are rich sources of antimicrobial compounds and drugs against several viral infections and diseases (da Silva Antonio et al., 2020; López-Malo et al., 2020). They are rich in natural antioxidants capable of reducing reactive oxygen species and reducing viral spread. Excess use of antimicrobials from synthetic sources may lead to the development of resistance (Usman et al., 2020) which can be avoided by using natural antimicrobials, derivatives, or mimics due to their diverse mechanisms of action (López-Malo et al., 2020). Plant-derived products selectively blocked the ACE 2 receptors (Benarba and Pandiella, 2020).

Specific natural compounds administered as a treatment were reported to show a recovery of 90% in some cases and prevented infections in healthy persons (Benarba and Pandiella, 2020). The Chinese traditional medicines adopted by Chinese health agencies are mixtures of different plants, and their parts consist of *Lonicera japonica*, *Scutellaria baicalensis*, and *Forsythia suspensa*; and *Ephedrae herba*, *Armeniacae semenamarum*, *Glycyrrhizae radix*, and *Gypsum fibrosum*, respectively and showed anti-SARS-CoV-2 activity (Yang et al., 2020). The plant and parts used were a rich source of metabolites and their mimics that improved health, and in some cases, the infection was wholly eradicated (Wang et al., 2020). Benarba and Pandiella (2020) suggested positive impacts of plant-derived products against SARS-CoV-2. Natural products were reported to inhibit the entry and replication of the virus. Motwalli and Alazmi (2021) suggested the effectiveness of natural zinc compounds (PubChem ID: 95372568 and 1,776,037) to inhibit the activity of SARS-CoV-2. Bioactive compounds originating from plants have been highlighted for their anti-SARS CoV2 activity (Ayivi et al., 2021). Among nucleoside analogs, remdesivir (an adenosine analog; Jorgensen et al., 2020), favipiravir (a guanine analog; Chen et al., 2020), and β-4-N4-hydroxycytidine (a ribonucleoside analog) inhibited SARS-CoV infections and replication by terminating RNA synthesis (Gordon et al., 2020) and activity (Chen et al., 2020).

Other traditional Chinese medicines approved for use include combinations of Chinese herbs. For example, Lianhuaqingwen with 11 herbs and *Scutellariae Radix* (dried roots of *Scutellariae baicalensis* Georgi) was found to effectively inhibit replication and activity of SARS-CoV, respectively (Wang et al., 2020). Naturally occurring flavonoid compounds in all 64 samples were evaluated. Among them, herbacetin, pectolinarin, and rhoifolin showed *in vitro* dose-dependent inhibition of SARS-CoV protease activity (Jo et al., 2020). *Rhizoma polygonati*, a Chinese herb, was evaluated against COVID-19 by Mu et al. (2021). A total of 10 active chemical compounds were identified, and all of them showed strong binding to the SARS-CoV virus. Two active compounds, diosgenin and (+)-syringaresinol-O-beta-D-glucoside, bound the protein better than ridasivir and lopinavir. Nebigil et al. (2020) evaluated flavaglines (cyclopental [b] benzofurans), which are found in several traditional Chinese medicines. The therapeutic potential of flavaglines was due to the regulation of the eukaryotic initiation factor 4A (*eIF4A*) and the inhibition of viral signaling. In addition, the flavaglines targeted proteins, helping to avoid issues of mutation and resistance. Computationally evaluated 36 flavonoids (with interaction energy of >−9 kcal/mol) from among 458 compounds against the SARS-CoV-2 main protease (Rameshkumar et al., 2021). Five compounds showed good bindings with all three types of targeted proteins and might be suitable inhibitors of SARS-CoV.

## Future Challenges of COVID-19 for Seafood Safety

The recent lockdowns imposed to control the pandemic have hampered aquaculture and fishing activities, causing an increase in demand and seafood prices, even though no study has shown any foodborne transmission of SARS-CoV-2. Some recent publications have highlighted the transmission of the virus through faulty processing conditions and procedures (Zuber and Brüssow, 2020). In addition, contaminated packaging materials have been a challenge for the food industry. However, no actual COVID has been shown to occur even if found on food or food packaging.

Seafood safety remains a challenging topic due to their susceptibility to spoilage requiring the maintenance of a chill and cold chain through processing, transportation, and storage. The seafood supply chain has a large number of links leading to many different risks. Several studies have shown that natural antimicrobials in ice and seafood can retard microbial growth, thus preventing deterioration. Natural antimicrobials also preserve the proteins and fats present in seafood. The latest help retain the improvements in immunity and the faster disease recovery associated with this seafood (Baptista et al., 2020) and prove the ability of seafood to build resilient food systems and sustainable healthy diets (Ahern et al., 2021).

## Conclusion

The recent COVID-19 pandemic caused by SARS-CoV-2, a highly pathogenic virus, has challenged everyone. Additionally, the pandemic has highlighted the importance of food, nutrition, and health. The seafood chain’s risk of transmitting COVID-19 is high due to the extensive exchange of hands. The available literature suggests high bioactive compounds found in seafood should be further explored for antiviral capacities. As processing and preservation of seafood are carried in cold temperature conditions, special care must be taken to monitor workers’ health and avoid the spread of COVID-19. The natural antimicrobials used as functional materials to preserve and enhance seafood should be evaluated for their ability to mitigate the risk of COVID-19 spread. The literature also discusses the importance of seafood, its authenticity, and its ability to supplement the development of a strong immunity to fight SARS-CoV-2 infections.

## Author Contributions

All authors listed have made a substantial, direct, and intellectual contribution to the work and approved it for publication.

## Funding

This work is supported by the PRIMA program under project BioProMedFood (ref. no. 2019-SECTION2-4 Project ID 1467). The PRIMA program is part of the European Union. This research was financially supported by the Scientific and Technological Research Council of Turkey (TUBITAK): N— UPAG 119N492 (PRIMA Programme Section 2). This publication was made possible by grant number NC.X-267-5-12-170-1 from the National Institute of Food and Agriculture (NIFA) and the Department of Family and Consumer Sciences and the Agriculture Research Station at North Carolina Agriculture and Technical State University (Greensboro, NC, United States). The authors extend appreciation to the International Scientific Partnership Program ISPP at King Saud University for funding this research work through ISPP-153.

## Conflict of Interest

The authors declare that the research was conducted in the absence of any commercial or financial relationships that could be construed as a potential conflict of interest.

## Publisher’s Note

All claims expressed in this article are solely those of the authors and do not necessarily represent those of their affiliated organizations, or those of the publisher, the editors and the reviewers. Any product that may be evaluated in this article, or claim that may be made by its manufacturer, is not guaranteed or endorsed by the publisher.

## Abbreviations

ACE 2: Angiotensin-converting enzyme
CDC: Centers for Disease Control and Prevention
COVID-19: Coronavirus disease 2019
EPA: Eicosapentaenoic
MAP: Modified atmosphere packaging
MERS-CoV: Middle east respiratory syndrome coronavirus
PLPRO: Target papain-like proteinase
RNA: Ribonucleic acid
SARS-CoV-2: Severe acute respiratory syndrome coronavirus 2
TMPRSS2: Transmembrane serine protease 2
WHO: World Health Organization
